# Cinacalcet Targets the Neurokinin-1 Receptor and Inhibits PKCδ/ERK/P65 Signaling to Alleviate Dextran Sulfate Sodium-Induced Colitis

**DOI:** 10.3389/fphar.2021.735194

**Published:** 2021-11-22

**Authors:** Yuehong Chen, Huan Liu, Qiuping Zhang, Yubin Luo, Liang Wu, Yutong Zhong, Zhigang Tang, Yaoyu Pu, Chenyang Lu, Geng Yin, Qibing Xie

**Affiliations:** Department of Rheumatology and Immunology, West China Hospital, Sichuan University, Chengdu, China

**Keywords:** cinacalcet, inflammatory bowel disease, neurokinin-1 receptor, tumor necrosis factor α, NF-κB

## Abstract

**Objective:** Inflammatory bowel disease is an immune-mediated chronic inflammatory disease of the gastrointestinal tract for which curative drugs are currently not available. This study was performed to assess the therapeutic effects of cinacalcet on dextran sulfate sodium (DSS)-induced colitis.

**Methods:** Primary macrophages obtained from bone marrow and the macrophage cell line RAW264.7 were used to examine the inhibitory effect of cinacalcet on cytokine production, the PKCδ/ERK/P65 signaling pathway, and NF-κB P65 translocation. Colitis was induced using DSS to assess the treatment effect of cinacalcet. Bioinformatics approaches were adopted to predict potential targets of cinacalcet, and a drug affinity responsive target stability (DARTs) assay was performed to confirm binding between cinacalcet and potential target.

**Results:** *In vivo* analysis showed that cinacalcet reduced the disease activity score, prevented shortening of the colon, diminished inflammatory cell infiltration, and protected the structural integrity of the intestinal wall. Cinacalcet also reduced production of the inflammatory cytokines TNFα, IL-1β, and IL-6 in the colon and sera of mice with DSS-induced colitis. *In vitro* studies revealed that cinacalcet suppressed the translocation of P65 and inhibited production of the inflammatory cytokines IL-1β and IL-6. Mechanistic studies revealed that the target of cinacalcet was neurokinin-1 receptor (NK1R) and their binding was confirmed by a DARTs assay. Furthermore, the inhibition of NK-κB P65 activation was found to occur via the suppression of PKCδ/ERK/P65 signaling mediated by cinacalcet.

**Conclusion:** Cinacalcet inhibits the activation of NF-κB and reduces the production of inflammatory cytokines by suppressing the PKCδ/ERK/P65 signaling pathway via targeting NK1R, suggesting that it can be used to treat inflammatory diseases, particularly colitis.

## Introduction

Inflammatory bowel disease (IBD) is an immune-mediated chronic inflammatory disease of the gastrointestinal tract and includes ulcerative colitis and Crohn’s disease. The incidence of IBD is increasing and varies among different geographical regions, with the highest annual incidence of 24.3/100,000 person-years for ulcerative colitis and 20.2/100,000 person-years for Crohn’s disease, creating a large economic burden on patients, their families, and healthcare systems (Molodecky et al., 2012; Windsor and Kaplan, 2019). Currently, the pathogenesis of IBD is unclear but it is considered to be associated with the genetic susceptibility of individuals, external environment, and intestinal mucosal immune system (Khor et al., 2011; Zhang and Li, 2014; de Souza and Fiocchi, 2016; Ramos and Papadakis, 2019). Tumor necrosis factor α (TNFα), which is at the top of the cytokine cascade, can be released by macrophages and stimulates the nuclear factor (NF)-κB signaling pathway which plays a crucial role in the pathogenesis of IBD (Neurath, 2014; de Souza and Fiocchi, 2016; Park and Jeen, 2018). After activation of the NF-κB signaling pathway, the transcription factor NF-κB P65 is activated and translocated from the cytoplasm to the nucleus, leading to synthesis of inflammatory cytokines including TNFα, interleukin (IL)-1β, and IL-6. Correspondingly, inhibition of TNFα-induced NF-κB activation shows therapeutic effects against IBD.

Currently, there is no cure for IBD, and current treatments can only control the symptoms, prevent disease progression, reduce complications, and improve the quality of life of patients (Sairenji et al., 2017). Traditional drugs for treating IBD include 5-aminosalicylic acid (5-ASA), salazosulfopyridine, and hormones. However, their effectiveness is insufficient, and they often cause adverse events. Nearly 50% of patients cannot reach remission and more than 40% of patients experience relapse. Glucocorticoids are associated with increased risks of infection, osteoporosis, and diabetes (Curkovic et al., 2013; Wang et al., 2016a; Murray et al., 2020). In recent years, biologics targeting molecules involved in inflammatory signaling pathways have shown potential for IBD treatment. However, they are costly and may increase the risk of infection and lymphoma development (Pithadia and Jain, 2011). Therefore, inexpensive and effective drugs with low risks of adverse events are urgently needed (Pithadia and Jain, 2011; Mao and Hu, 2016). Currently, repurposing drugs that are already in clinical use is a time-saving and cost-effective approach for identifying new drugs to treat IBD (Chong and Sullivan, 2007).

Cinacalcet, a calcimimetic type Ⅱ compound, is widely used to treat primary and secondary hyperparathyroidism by targeting the calcium-sensing receptor (CaSR) to reduce the production of parathyroid hormone. The main adverse events of cinacalcet are mild to moderate nausea and vomiting, the frequency of which can be reduced when vitamin D analogs are concomitantly used. In clinical studies, the adverse events caused by cinacalcet were comparable to those in the placebo-treated group (de Francisco, 2005; Messa et al., 2008; Junaid and Patel, 2020). A previous study suggested that Cinacalcet can be used as an add-on therapy for IBD to reduce production of the cytokines interferon-γ, IL-1α, and TNFα in the colon of dextran sulfate sodium (DSS)-induced colitis BALB/c mice (Elajnaf et al., 2019). Here, we examined whether cinacalcet is effective for attenuating the severity of DSS-induced colitis by inhibiting the activity of NF-κB in C57BL/6 mice.

## Materials and Methods

### Mice

Seven-week-old wild-type C57BL/6 mice were obtained from Beijing Huafukang Biotechnology Company (Beijing, China). The mice were acclimatized to the Animal Facility of Chengdu Frontier Medical Center, West China Hospital, Sichuan University, for 1 week and then used in the experiments. The animal facility is specific pathogen-free. The mice were randomly assigned to cages, with five mice in each cage. The mice had free access to food and water and were maintained on a 12-h/12-h light/dark cycle at a constant temperature of 22–24°C.

All animal experiments were conducted following protocols approved by the Animal Ethics Committee of West China Hospital, Sichuan University (Nos. 2020243A and 20211318A).

### Reagents and Materials

Dulbecco’s modified Eagle’s medium (DMEM, 10-017-CM) was purchased from Corning, Inc. (Corning, NY, United States) and fetal bovine serum (10099-141) was purchased from Gibco (Grand Island, NY, United States). DSS (molecular weight: 36,000–50,000, 160110) was purchased from MP Biomedicals (Santa Ana, CA, United States). TNBS (Picrylsulfonic acid solution, P2297) was from Sigma-Aldrich (Milwaukee, WI, United States), and 5-aminosalicylic acid (5-ASA, HY-15027), cinacalcet (HY-70037), NPS-2143 (a selective and potent CaSR antagonist, HY-10007), and JSH-23 (an NF-κB inhibitor, HY-13982) were purchased from MedChemExpress (Monmouth Junction, NJ, United States). Plastic feeding tubes (TFEP-001, 2.25 × 50 mm) were from Shanghai Yuyan Instruments Company (Shanghai, China). The RNeasy^®^ Mini kit (74104) was purchased from Qiagen (Hilden, Germany). The secondary fluorescence antibody (Alexa Fluor^®^ 488, ab150077; Alexa Fluor^®^ 647, ab150083), recombined TNFα protein (ab9642), anti-GAPDH antibody (EPR16891, ab181602), glycerol mounting medium with DAPI (ab188804), and mammalian cell lysis buffer (5×, ab179835) were from Abcam (Cambridge, UK). ChamQ SYBR qPCR Master Mix (Q311-02) and HiScript^®^ Ⅲ RT SuperMix for qPCR (+gDNA wiper, R323-01) were purchased from Vazyme (Nanjing, China). The NF-κB P65 antibody (AF5006), phospho-NF-κB antibody (S536, AF 2006), extracellular signal-regulated kinase (ERK) antibody (AF0155), phosphorylated (p)-ERK antibody (AF1015), histone H3 antibody (AF0863), enhanced chemiluminescence kit (KF005), PKCδ antibody (AF6408), p-PKCδ antibody (Thr645, AF3408), and NK1R antibody (DF4996) were from Affinity Biosciences (Cincinnati, OH, United States). The myeloperoxidase peroxidation activity fluorometric assay kit (E-BC-F013) was from Elabscience (Wuhan, China). The F4/80 monoclonal antibody (BM8) (14-4801-82) and NE-PER nuclear and cytoplasmic extraction reagents (78833) were from ThermoFisher Scientific (Waltham, MA, United States). Pronase from *Streptomyces griseus* (C756W53, 10165921001) was purchased from MilliporeSigma (Temecula, CA, USA). The Immobilon^®^-P transfer membrane, 0.45-μm (IPVH00010), was purchased from Merck Millipore (Billerica, MA, United States). Macrophage colony-stimulating factor (M-CSF, 576406) was from Biolegend (San Diego, CA, USA). The mouse IL-1β enzyme-linked immunosorbent assay (ELISA) kit (E08054M), mouse IL-6 ELISA kit (E04639M), and mouse TNFα ELISA kit (E04741M) were from CUSABIO (Houston, TX, United States). Cell lysis buffer (P0013) and the BCA protein quantitation assay (P0010) were purchased from Beyotime (Shanghai, China). The protease inhibitor cocktail (GK10014), phosphatase inhibitor cocktail Ⅰ (GK10011), and phosphatase inhibitor cocktail Ⅱ (GK10012) were from Glpbio (Montclair, CA, United States). PBS (1×, G4202) was purchased from Servicebio (Wuhan, China).

### Real-Time Quantitative PCR

The total RNA was extracted using a RNeasy mini kit and cDNA was synthesized using HiScript^®^ Ⅲ RT SuperMix. ChamQ SYBR qPCR Master Mix was used to perform real-time quantitative PCR (qRT-PCR) on a CFX96™ Real-Time system (Bio-Rad, Hercules, CA, United States). The mRNA expression level was calculated using the 2^−ΔΔCT^ method, and fold-changes in target mRNA levels were normalized to that of *GAPDH*. The following specific SYBR primers were used for target gene amplification: mouse *TNFα* (5′-3′) F: CTG TAG CCC ACG TCG TAG C, R: TTG AGA TCC ATG CCG TTG; mouse *IL-1β* (5′-3′) F: AAT CTC ACA GCA GCA CAT CA, R: AAG GTG CTC ATG TCC TCA TC; mouse *IL-6* (5′-3′) F: TTC CAT CCA GTT GCC TTC TTG, R: AGG TCT GTT GGG AGT GGT ATC; mouse *CCL2* (5′-3′) F:TAG CAG CCA CCT TCA TTC, R: CTT GGG GTC AGC ACA GAT; mouse *IL-8* (5′-3′) F: CCT GCT TGA ATG GCT TGA ATA C, R: GGT GTC CTG ATT ATC GTC CTC; mouse *GAPDH* (5′-3′) F: AGA ACA TCA TCC CTG CAT CC, R: AGT TGC TGT TGA AGT CGC.

### Western Blotting

Proteins were extracted by lysing the cells, and the protein concentration was determined by BCA assay. The samples were then heated for 10 min at 95°C after adding loading buffer, separated on a sodium dodecyl sulfate-polyacrylamide gel, and transferred onto a membrane using a wet transfer system. The membrane was blocked with 5% (w/v) non-fat milk in Tris-buffered saline containing Tween 20 (0.1%) for 30 min at room temperature, followed by incubation with primary antibodies overnight at 4°C and secondary antibody for 1 h at room temperature. The bands on the membrane were developed using the enhanced chemiluminescent kit.

### Immunofluorescence Staining

RAW264.7 cells were seeded onto glass cover slips in 24-well plates. After incubation in DMEM supplemented with 1% FBS and 1% penicillin–streptomycin overnight and addition of cinacalcet (5 μM) for 2 h, TNFα (10 ng/ml) was added to the cells, and incubated for 4 h. After fixation in 4% formaldehyde for 10 min, incubation in 0.1% Triton X-100 for 5 min, and blocking with 20% donkey serum for 1 h at room temperature, the samples were incubated with the primary antibody against P65 at a 1:100 dilution in blocking buffer at 4°C overnight. The next day, the samples were incubated with fluorescence-conjugated secondary antibody (1:400) for 1 h at 37°C in the dark, mounted with DAPI, and sealed with transparent nail polish.

Colon tissue paraffin sections (5 μm) were dewaxed in xylene and hydrated in gradient ethyl alcohol. After antigen retrieval using 0.1% trypsin diluted with 0.1% calcium chloride (w/v) at 37°C for 30 min, slides were blocked with 20% goat serum for 1 h at room temperature, followed by incubation with the primary antibody against F4/80 at a 1:100 dilution in blocking buffer at 4°C overnight. The next day, slides were incubated with fluorescence-conjugated secondary antibody (1:400) for 1 h at 37°C in the dark, mounted with DAPI, and sealed with transparent nail polish.

Images were captured using a ZEISS positive fluorescence microscope (AX10 imager A2, Germany), and the merging of images was performed using ImageJ (1.48v, United States).

### Enzyme-Linked Immunosorbent Assay

Levels of TNFα, IL-1β, and IL-6 in cell culture supernatants, colon tissue, and mouse sera were detected using mouse ELISA kits according to the manufacturer’s instructions. Optical density was measured with a microplate reader (CLARIOstar, BMG LABTECH, German) at a wavelength of 450 nm, and target molecule concentrations were determined using the standard curve. The colon tissues from mice (1 mg) were placed in 10 μL PBS supplemented with protease inhibitor cocktail (1:100) and then homogenized with a PowerLyzer 24 (Qiagen). Homogenates were stored at −20°C overnight, and then, cells were lysed by repeated freeze-thawing, three times in liquid nitrogen. The samples were then centrifuged at 4 °C for 10 min at 12,000 ×*g*, and the supernatants were collected to detect cytokine levels.

### Myeloperoxidase Activity

Colons were removed and kept in liquid nitrogen. Myeloperoxidase (MPO) activity in colon tissue was measured using a MPO assay kit according to manufacturer’s instructions. To prepare tissue homogenates, colon tissues were weighed accurately and homogenized according to the volume ratio of 1 g:9 ml homogenate media. The homogenates were centrifuged 10,000 ×*g* at 4°C for 10 min. Then, supernatants were collected, and MPO activity was assessed. Data were collected based on an excitation of 535 nm and emission of 587 nm, as detected by a spectrophotometer (CLARIOstar, BMG LABTECH, German). Protein concentrations of homogenate supernatants were detected with a BCA protein assay kit. Results were normalized to protein concentrations.

### Isolation of Bone Marrow-Derived Macrophages

Bone marrow-derived macrophages (BMDMs) originated from normal wild-type C57BL/6 mice. After sacrificing the mice via cervical dislocation, the femur and tibia were separated. Both ends of the bones were cut to expose the bone medullary cavity and then centrifuged to collect the bone marrow cells at 13,000 ×*g* for 60 s. The cells were seeded into plates containing M-CSF (10 ng/ml) in DMEM for 3 days, after which the culture medium was replaced with fresh DMEM containing M-CSF at 10 ng/ml for another 3 days; the cells were then used in the experiments.

### Prediction of Cinacalcet Binding Target

The TargetNet website (http://targetnet.scbdd.com), an open website for drug target prediction, was used to find the possible binding target of cinacalcet, and prediction results showed that cinacalcet had a 98.8% possibility of binding to the target neurokinin-1 receptor (NK1R). To visualize the binding between cinacalcet and NK1R, a 3D structure, originated from human, showing binding sites of cinacalcet with respect to NK1R was obtained from the COACH-D website (Wu et al., 2018) (https://yanglab.nankai.edu.cn/COACH-D/), a professional website for the prediction of ligand-binding sites via a molecular docking method, which was developed by professor Jianyi Yang from Nankai University.

### Drug Affinity Responsive Target Stability Assay

Drug affinity responsive target stability (DARTs) experiments were performed to test the binding between cinacalcet and NK1R based on previously reports (Lomenick et al., 2009; Pai et al., 2015). Briefly, RAW264.7 cells were lysed in lysis buffer (1× mammalian cell lysis buffer) on ice for 10 min. Lysates were collected in a clean pre-chilled 1.5 ml tube and centrifuged at 18,000 ×*g* for 10 min at 4°C, and then, the supernatants, which were the cell lysates, were transferred to a new 1.5 ml tube. After measuring the protein concentration by performing a BCA assay, 99 μL of cell lysates with 1 μL DMSO or 1 μL cinacalcet at 100 mM were mixed and incubated on a shaker for 30 min at room temperature. Then, the mixtures of cell lysates with DMSO or cinacalcet were equally distributed into four tubes, and pronase was added at concentrations of 1:100, 1:200, and 1:400 based on the ratios of pronase to total protein in each tube for 5 min at room temperature. Digestion was stopped by adding 20X protease inhibitor cocktail, and samples were incubated on ice for 10 min, followed by the addition of SDS-PAGE loading buffer and boiling for 10 min at 95°C; then, SDS-PAGE was performed.

### DSS Induced Colitis Model

The DSS-induced colitis model was established by supplying 8-week-old C57BL/6 mice with drinking water containing 3% DSS for 5 days followed by normal drinking water for 3 days (Wirtz et al., 2007; Wei et al., 2014). Model mice were grouped into a vehicle group, 5-ASA group (Funakoshi et al., 2012; Yamada et al., 2014) (50 mg/kg, serving as a positive control), and 0.1 mg/kg, 1 mg/kg, and 10 mg/kg cinacalcet groups, with 10 mice in each group, five female and five male mice. Cinacalcet and 5-ASA dissolved in DMSO were aliquoted and stored at −80°C (for up to 3 months). At the time of experiment, drugs in DMSO were diluted by in a solution of water:ethanol:2% acetic acid, at 8:3:1 (v/v), making the final volume of DMSO less than 5%. Drugs were administered by oral gavage 3 days before 3% DSS drinking water was started until the mice were sacrificed. Weight loss, stool consistency, and rectal bleeding were recorded each day starting from the day of 3% DSS water supplementation until the end of the experiment, and the disease activity index was calculated as the sum of the scores for weight loss, stool consistency, and rectal bleeding. A higher score suggested a more serious condition (Wang et al., 2016b): body weight loss (0 ≤ 5%, 1 = between 5 and 10%, 2 = between 10 and 15%, 3 = between 15 and 20%, 4 = >20%), stool consistency (0 = normal, 2 = loose stool, 4 = diarrhea), rectal bleeding (0 = negative, 2 = blood trace, 4 = gross blood). At the end of the experiment, the mice were sacrificed, sera and colon were collected. The colon length was measured with a digital caliper (0–150 mm, DEGUQMNT, Shanghai Meinite Industrial Company, Shanghai, China).

### TNBS-Induced Colitis Model

To establish a TNBS-induced IBD model (Wirtz et al., 2007), 8-week-old C57BL/6 mice were pre-sensitized by adding 150 μL 1% TNBS on the back of two forearms after moulting. Five days later, the mice were grouped in the following groups: vehicle, 5-ASA (50 mg/kg), 0.1 mg/kg cinacalcet, 1 mg/kg cinacalcet, and 10 mg/kg cinacalcet, with 10 mice in each group (half male and half female). Drugs were orally delivered every day until the end of the experiment. After drug pre-treatment for 2 days, mice were used to establish the TNBS-induced colitis model via the injection of 100 μL 3% TNBS into the colon through the anus, and body weight was recorded every day thereafter. Four days after modeling, mice were sacrificed and sera and colons were collected.

### Hematoxylin and Eosin Staining and Quantitative Analysis

Hematoxylin and eosin (H&E) staining was performed as follows. Briefly, after deparaffinization and rehydration, the slides were treated with hematoxylin, clarifier, blue buffer, and eosin phloxine, followed by dehydration using graded ethanol. The H&E stained sections were scanned by the automated quantitative pathology imaging system (Vectra Polaris, United States).

The H&E staining score of the colon (Erben et al., 2014) was calculated as the sum of inflammatory cell infiltration and intestinal wall structure integrity, with higher scores indicating more serious inflammation. The scores of inflammatory cell infiltration were as follows: 0 = normal, 1 = inflammatory cell only infiltrated the mucosa, 2 = inflammatory cell reached the mucosa and sub-mucosa, 3 = inflammatory cell detected throughout the intestinal wall. The scores for the structural integrity of the intestinal wall were assessed as the change in epithelial cells: 0 = normal, 1 = inflammatory cells were locally infiltrated, 2 = focally formed ulceration, 3 = extensively formed ulceration with or without granulation tissue or pseudo-polyps.

### Statistical Analysis

Data were organized and analyzed using GraphPad Prism software version 8.0 (GraphPad, Inc., La Jolla, CA, United States) and SPSS software version 22 (SPSS, Inc., Chicago, IL, United States), and the statistical significance of differences among groups was determined by one-way analysis of variance and Bonferroni post-hoc tests. All data are expressed as the mean with standard error of the mean, and *p* < 0.05 was considered to indicate statistically significant results.

## Results

### Cinacalcet Inhibits the Production of Cytokines Induced by TNFα

**FIGURE 1:**
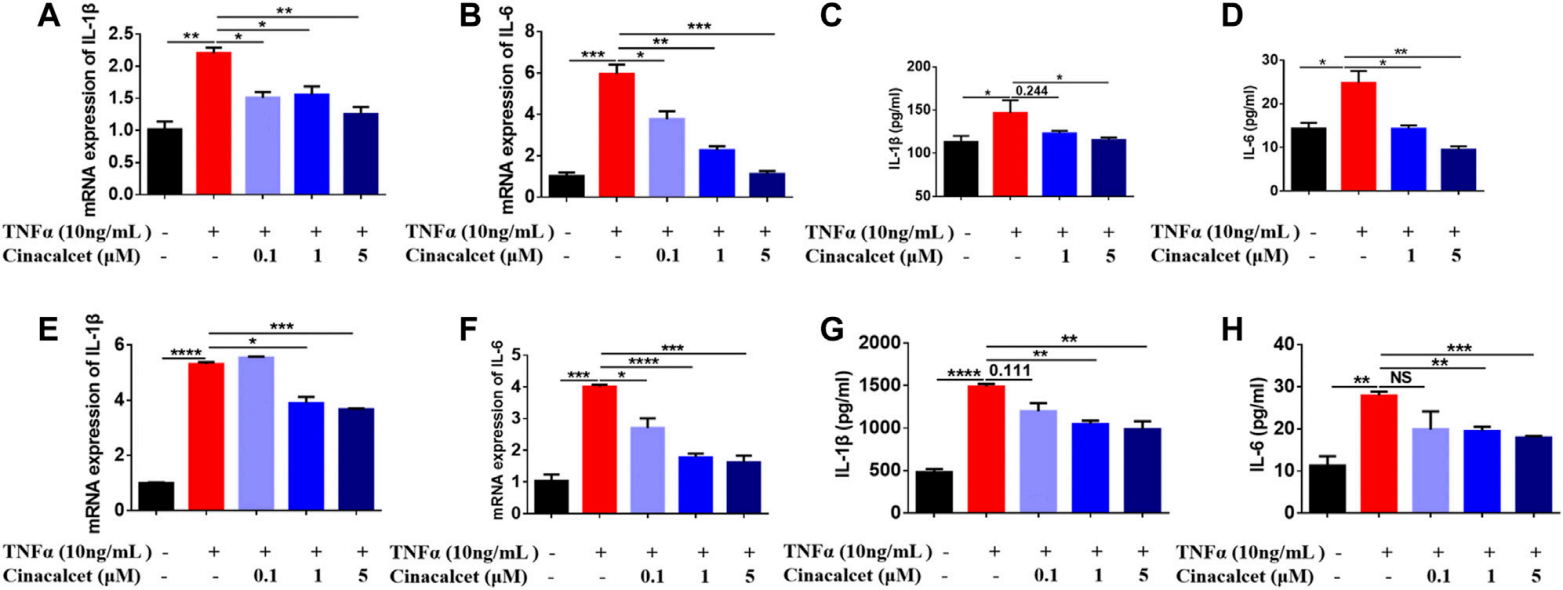
Cinacalcet inhibits the production of cytokines induced by TNFα. Bone marrow-derived macrophages (BMDMs) or RAW264.7 cells were treated with TNFα (10 ng/ml) in the absence or presence of cinacalcet (0.1, 1, 5 μM) for 24 h. mRNA expression levels of *IL-1β* and *IL-6*, normalized to the internal reference *GAPDH*, were detected by qRT-PCR. Cytokine levels in the supernatants were detected by ELISA. **(A, B)** mRNA expression levels of *IL-1β* and *IL-6* in RAW264.7 cells. **(C, D)** Cytokine levels of IL-1β and IL-6 in supernatants of RAW264.7 cells. **(E, F)** mRNA expression levels of *IL-1β* and *IL-6* in BMDMs. **(G, H)** Cytokine levels of IL-1β and IL-6 in supernatants of BMDMs. *p < 0.05, **p < 0.01, ***p < 0.001, ****p < 0.0001. Data are shown as the mean with standard deviation. Three independent experiments were performed.

TNFα is a well-known pro-inflammatory cytokine that can activate NF-κB and induce the production of inflammatory cytokines, such as IL-1β and IL-6. To test whether cinacalcet has an inhibitory effect on the production of inflammatory cytokines induced by TNFα, we used both the primary macrophages BMDMs and the macrophage cell line RAW264.7. In RAW264.7 cells, TNFα increased the mRNA expression levels of the inflammatory cytokines *IL-1β* and *IL-6* by 2- and 6-fold, respectively, whereas cinacalcet significantly and dose-dependently reduced their expression (Figures 1A,B). The secretion levels of IL-1β and IL-6 in the cell culture supernatants induced by TNFα were also dose-dependently decreased by cinacalcet (Figures 1C,D). Similarly, in BMDMs, TNFα clearly increased the mRNA expression and secretion levels of IL-1β and IL-6, whereas cinacalcet decreased TNFα-induced the mRNA expression and secretion of IL-1β and IL-6 in a dose-dependent manner (Figures 1E–H). Collectively, cinacalcet suppressed the production of inflammatory cytokines in macrophages.

### Cinacalcet Shows Therapeutic Effects Against DSS-Induced Colitis

**FIGURE 2:**
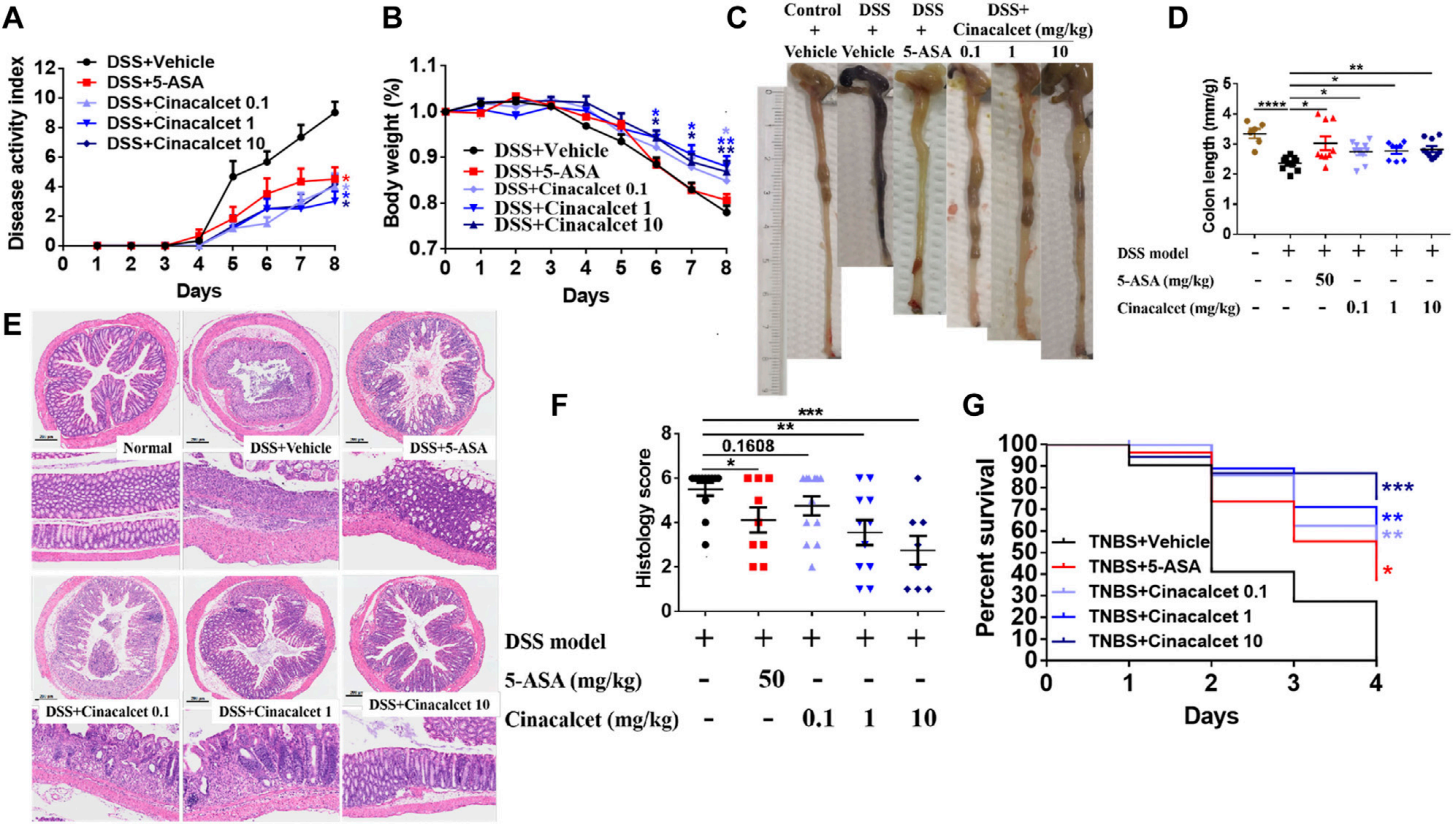
Cinacalcet shows therapeutic effects against dextran sulfate sodium (DSS)-induced colitis. A DSS-induced colitis model, based on 8-week-old wild-type C57BL/6 mice, was established via free access to drinking water containing 3% DSS for 5 days followed by normal drinking water for 3 days, with 10 mice in each group. Drug dilution buffer for the vehicle group, 5-ASA (positive control, 50 mg/kg), and cinacalcet (0.1, 1, 10 mg/kg) were orally delivered daily 3 days before administering 3% DSS drinking water until the mice were sacrificed. **(A)** Disease activity index score. **(B)** Body weight. **(C)** Representative colon figures in each group. **(D)** Statistic colon lengths of each group. **(E)** H&E staining (200 μm). **(F)** Quantification of histology score of H&E staining. **(G)** Percent survival in TNBS-induced colitis model (*n* = 10). *p < 0.05, **p < 0.01, ***p < 0.001, ****p < 0.0001.

As TNFα, IL-1β, and IL-6 play crucial roles in the pathogenesis of colitis and cinacalcet suppressed the production of those inflammatory cytokines, we assessed the anti-inflammatory effect of cinacalcet on DSS-induced colitis. Low (0.1 mg/kg), medium (1 mg/kg), and high (10 mg/kg) doses were used to test the treatment effect of cinacalcet on DSS-induced colitis, with 5-ASA used as a positive control. The disease activity index score, which is the sum of the scores of body weight loss, stool consistency, and rectal bleeding, ranges from 0 to 12, with a higher score indicating more severe disease. This began to increase on day 3 after model establishment and was obviously increased on subsequent days in the DSS-induced colitis mouse model (Figure 2A). In contrast, 5-ASA and cinacalcet at the three doses significantly reduced the severity of DSS-induced colitis, as indicated by considerably reduced disease activity index scores (Figure 2A). Cinacalcet at the three doses prevented body weight loss in DSS-induced colitis (Figure 2B). Regarding colon length, 5-ASA and cinacalcet at the three doses prevented colon shortening, a feature of colitis (Figures 2C,D). H&E staining of paraffin-embedded sections of the colon tissue (5-μm) was performed to assess inflammatory cell infiltration and intestinal wall structural integrity; inflammatory cells infiltrated the mucosa, sub-mucosa, and intestinal wall, and the cell wall structural integrity was disrupted in mice with DSS-induced colitis treated with the buffer used to dilute the drug (Figures 2E,F). Treatment with cinacalcet considerably alleviated the infiltration of inflammatory cells and protected the structural integrity of the intestinal wall (Figures 2E,F). These data suggest that cinacalcet attenuates the severity of DSS-induced colitis in mice. We also assessed the treatment effect of cinacalcet on TNBS-induced colitis, and results showed that on the fourth day after model establishment, all mice were dead in the TNBS model group treated with the buffer used to dilute the drug, whereas both 5-ASA and cinacalcet at three doses reduced the mortality, showing a protective effect for cinacalcet on TNBS-induced colitis (Figure 2G).

### Cinacalcet Inhibits Inflammatory Cytokine Production in the Colon With DSS-Induced Colitis

**FIGURE 3:**
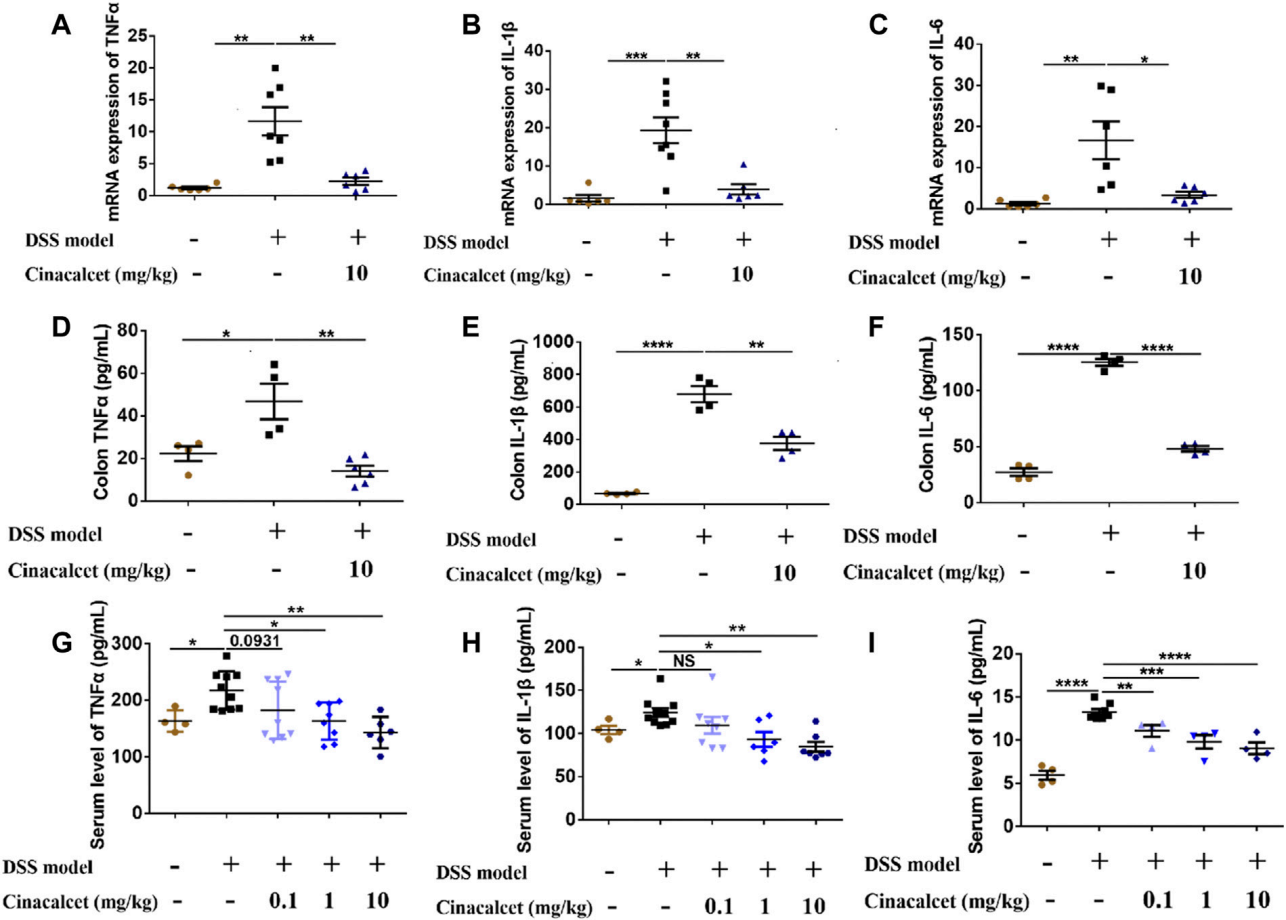
Cinacalcet inhibits inflammatory cytokine production in dextran sulfate sodium (DSS)-induced colitis. The expression and secretion of cytokines in cinacalcet-treated mice were tested by qRT-PCR and ELISA. **(A–C)** mRNA expression levels of *IL-1β*, *IL-6*, and *TNFα* in the colon tested by qRT-PCR. **(D–F)** Secretion levels of IL-1β, IL-6, and TNFα in the colon detected by ELISA. **(G–I)** Serum levels of IL-1β, IL-6, and TNFα detected by ELISA. *p < 0.05, **p < 0.01, ***p < 0.001, ****p < 0.0001. Data are shown as the mean with standard deviation.

Previous studies on DSS-induced colitis showed that inflammatory cytokines are significantly increased and play important roles in the pathogenesis of the disease (Kim et al., 2012; Neurath, 2014). We found that cinacalcet inhibited the production of inflammatory cytokines *in vitro* and attenuated the severity of DSS-induced colitis *in vivo*. To investigate whether cinacalcet suppresses the production of inflammatory cytokines in DSS-induced colitis, we detected the mRNA expression and secretion levels of cytokines in the colon by qRT-PCR and ELISA. The mRNA expression levels of TNFα, IL-1β, and IL-6 were increased by nearly 12-, 18-, and 16-fold, respectively, and the secretion levels of TNFα, IL-1β, and IL-6 were elevated by approximately 2-, 10-, and 3-fold, respectively, in DSS-induced colitis. Cinacalcet significantly reduced both the expression and secretion of TNFα, IL-1β, and IL-6 in the colons of mice with DSS-induced colitis (Figures 3A–F). Similarly, cinacalcet also reduced the release of cytokines, namely TNFα, IL-1β, and IL-6, in the sera (Figures 3G–I). Collectively, cinacalcet reduces production of the inflammatory cytokines TNFα, IL-1β, and IL-6 in a DSS-induced colitis model.

### Cinacalcet Reduces Inflammatory Cell Infiltration in a DSS-Induced Colitis Model

**FIGURE 4:**
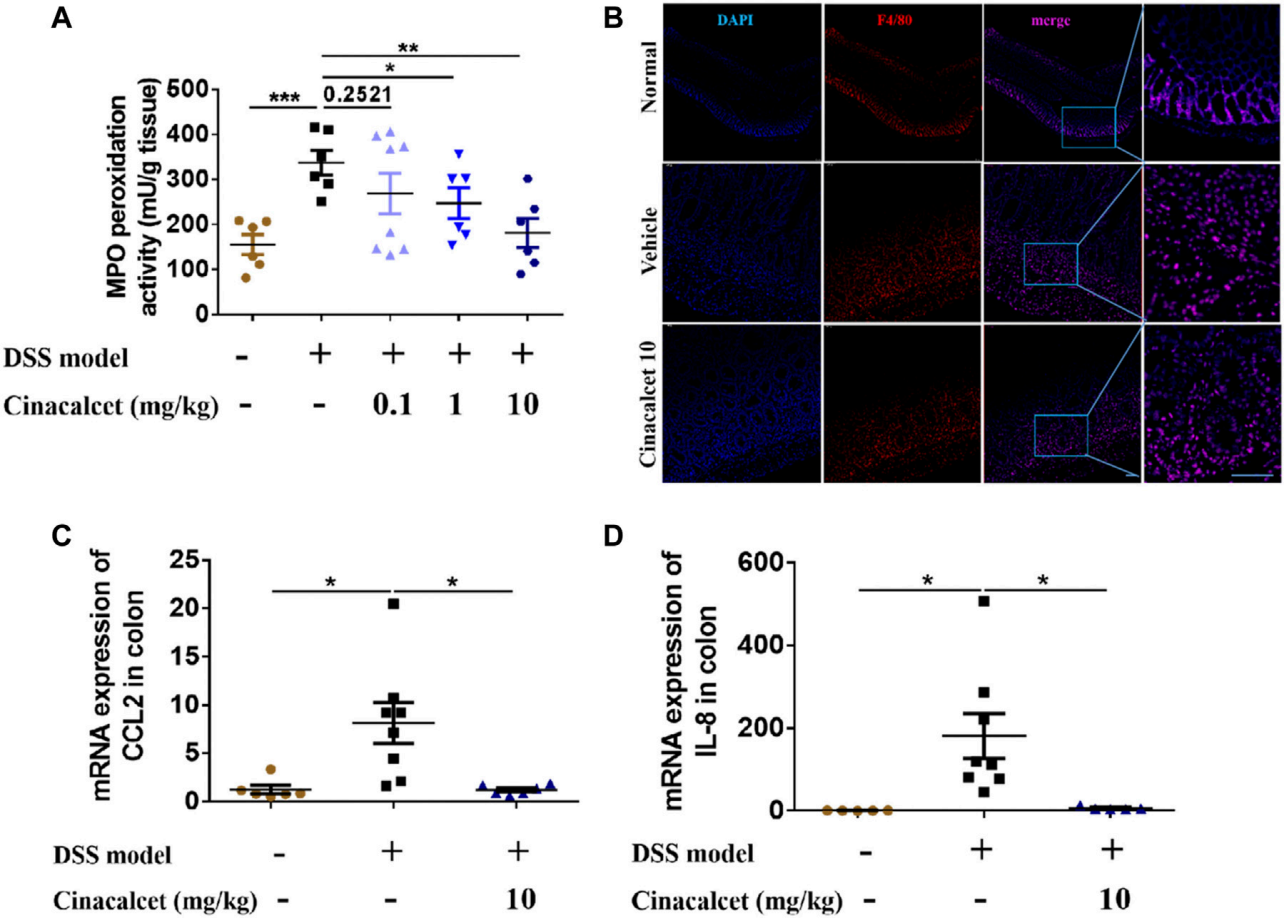
Cinacalcet redcues inflammatory cell infiltration in dextran sulfate sodium (DSS)-induced colitis model. **(A)** Myeloperoxidase (MPO) activity was assessed to quantify the infiltration of neutrophils in the colon. **(B)** immunofluorescence was conducted to show the infiltration of macrophages in the colon (50 μm). **(C, D)** qRT-PCR was performed to detect the mRNA expression levels of chemokines *CCL2* and *IL-8* in the colon. *p < 0.05, **p < 0.01, ***p < 0.001. Data are shown as the mean with standard deviation.

Based on the H&E staining, inflammatory cells substantially infiltrated the entire intestinal wall of the colon in mice with DSS-induced colitis, whereas inflammatory cell infiltration was significantly reduced in cinacalcet-treated mice. We further performed MPO activity assays and immunofluorescence staining to test the infiltration of neutrophils and macrophages. Results of MPO activity detection showed that MPO activity was significantly increased in the DSS-induced colitis mice, which was obviously inhibited by treatment with cinacalcet (Figure 4A), and immunofluorescence staining of macrophages suggested that cinacalcet also reduced macrophage infiltration (Figure 4B). Monocyte chemoattractant protein-1 (MCP-1, also called CCL2) is a potent macrophage chemoattractant, whereas macrophage inflammatory protein-2 (MIP-2) is a murine homolog to human of IL-8, which has the function of attracting neutrophils (Baggiolini and Clark-Lewis, 1992; Wetzler et al., 2000; Kanda et al., 2006). Therefore, we further investigated whether the decreased inflammatory cell infiltration was due to the reduced mRNA expression of chemokines *CCL2* and *IL-8.* Results showed that cinacalcet could reduce the expression of both (Figures 4C,D). Collectively, cinacalcet reduced inflammatory cell infiltration in the colon.

### Cinacalcet Suppresses the Activity of NF-κB

**FIGURE 5:**
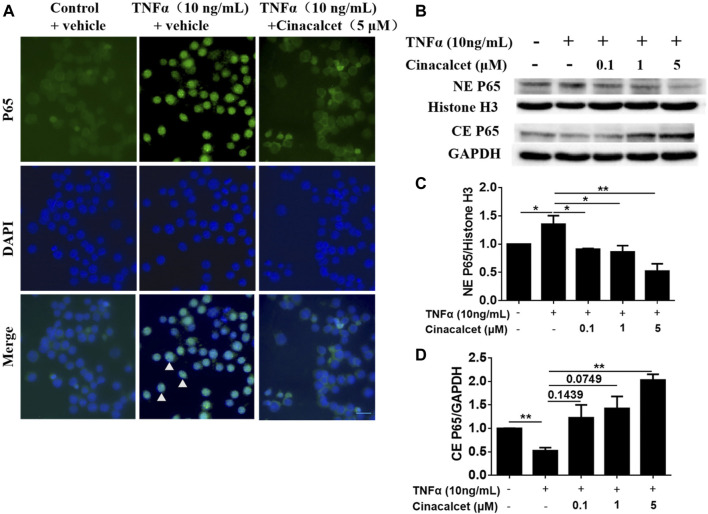
Cinacalcet inhibits the translocation of P65 from the cytoplasm to the nucleus. **(A)** RAW264.7 cells were starved with 2% FBS overnight. After treating the cells with cinacalcet (5 μM) for 2 h, TNFα (10 ng/ml) was added for 4 h. Immunofluorescence of P65 was performed (20 μm). White arrow heads indicate the aggregation of P65 in the nuclei. **(B)** Bone marrow-derived macrophages (BMDMs) were seeded in 10 cm plates. After starvation with 2% FBS overnight, the cells were treated with cinacalcet (0.1, 1, 5 μM) for 2 h followed by TNFα (10 ng/ml) for 45 min. Cytoplasmic and nuclear proteins were extracted for western blotting. **(C)** Quantification of NE P65 expression standardized to the internal expression of histone H3. **(D)** Quantification of CE P65 expression standardized to the internal expression of GAPDH. *p < 0.05, **p < 0.01. Data are shown as the mean with standard deviation. Three independent experiments were performed.

As cinacalcet inhibited the production of inflammatory cytokines and TNFα-stimulated NF-κB activity plays a crucial role in the pathogenesis of IBD, we further tested whether the suppression of cytokine production mediated by cinacalcet occurred via NF-κB activity inhibition. Thus, we investigated the effect of cinacalcet on P65 translocation by immunofluorescent staining and western blotting. Positive staining of P65 was observed in the nucleus after stimulation with TNFα, whereas P65 was mainly observed in the cytoplasm after treatment with cinacalcet, indicating that cinacalcet inhibited the translocation of P65 from the cytoplasm to the nucleus (Figure 5A). Results of western blotting showed that P65 expression in the cytoplasm was reduced by nearly 50% after TNFα stimulation, as this activation led to translocation of P65 into the nucleus; correspondingly, the expression of P65 in the nucleus was increased by approximately 30% (Figures 5B–D). In contrast, the expression of P65 in the cytoplasm was dose-dependently increased after treatment with cinacalcet, as cinacalcet prevented the activation of NF-κB and thus inhibited the translocation of P65 from the cytoplasm to the nucleus, and P65 expression in the nucleus was correspondingly reduced after treatment with cinacalcet (Figures 5B–D). Based on these results, cinacalcet inhibited the activity of NF-κB.

### Cinacalcet Suppresses the PKCδ/ERK/P65 Signaling Pathway

**FIGURE 6:**
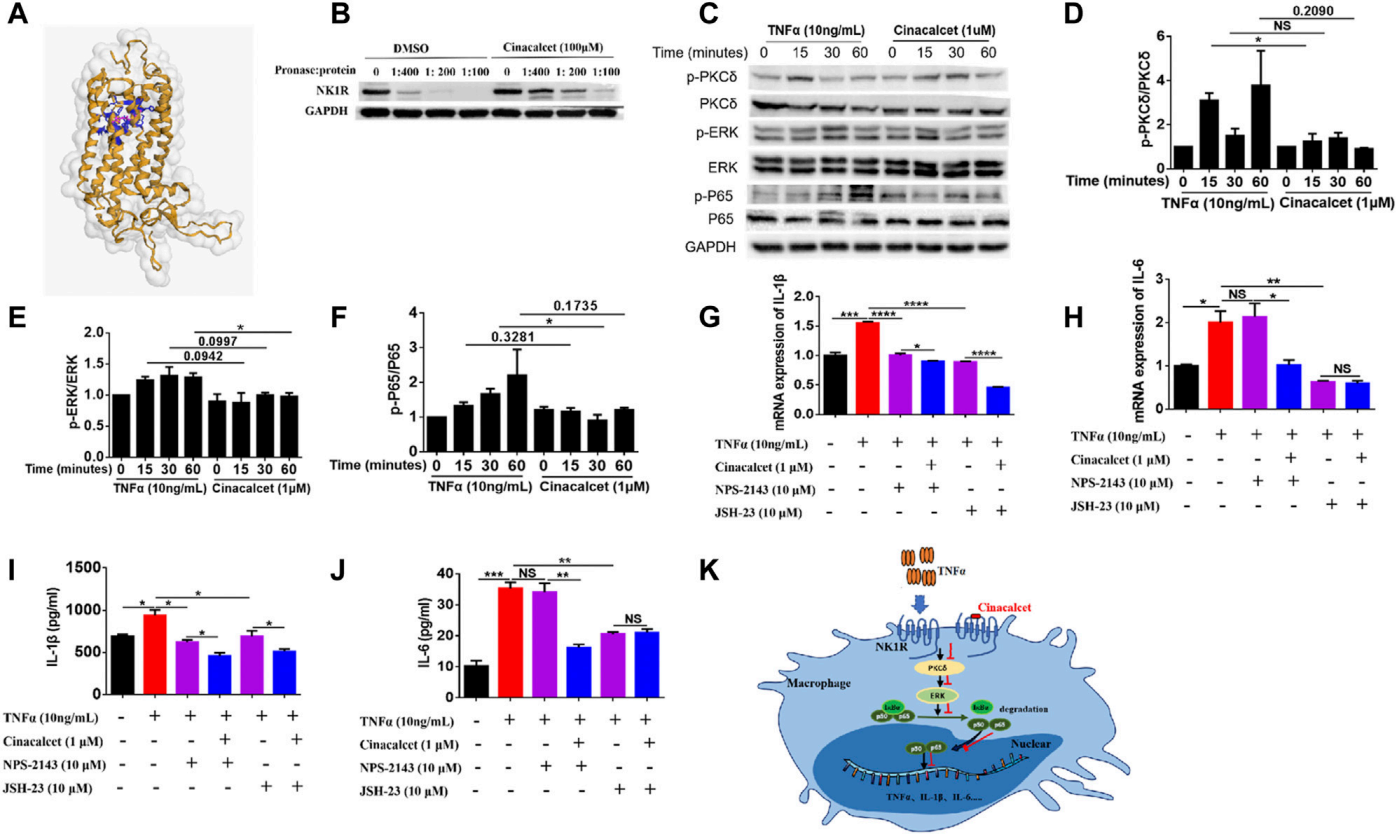
Cinacalcet suppresses the PKCδ/ERK/P65 signaling pathway. **(A)** Visualization of predicted the binding sites of cinacalcet with respect to NK1R. Structures on the yellow color and grey surface represent the NK1R structure, blue structures represent the amino acids of cinacalcet that bind to NK1R, and structures in pink represent structures of cinacalcet. **(B)** DARTs assay to test the binding of NK1R and cinacalcet. **(C)** Representative western blot results to show that cinacalcet suppresses the PKCδ/ERK/P65 signaling pathway. Bone marrow-derived macrophages (BMDMs) were starved with 2% FBS overnight. After treating the cells with cinacalcet (1 μM) for 2 h, TNFα (10 ng/ml) was added for different periods (0, 15, 30, 60 min). Proteins were extracted from the cells, and western blotting was performed. **(D)** Quantification of p-PKCδ expression. **(E)** Quantification of p-ERK expression. **(F)** Quantification of p-P65 expression. **(G, H)** qRT-PCR was performed to test the mRNA expression levels of *IL-1β* and *IL-6* in BMDMs after stimulation with TNFα (10 ng/ml) in the presence or absence of cinacalcet for 24 h **(I, J)** ELISA was performed to detect IL-1β and IL-6 levels in BMDM supernatants after stimulation with TNFα (10 ng/ml) in the presence or absence of cinacalcet for 24 h. **(K)** Proposed model explaining the anti-TNF activity of cinacalcet through direct targeting of the NK1R pathway. *p < 0.05, **p < 0.01, ***p < 0.001, ****p < 0.0001. Data are shown as the mean with standard deviation. Three independent experiments were performed.

To figure out how cinacalcet inhibited the activation of NF-κB, we used bioinformatics to predict its target and identified NK1R; further their binding was visualized based on a 3D structure (Figure 6A). To further confirm their binding, we performed DARTs, and results showed that cinacalcet could protect NK1R from degradation by pronase, suggesting its binding to NK1R (Figure 6B). A previous study reported that NK1R could mediate PKCδ/ERK/P65 signaling (Spitsin et al., 2018), and thus, we performed western blotting to test whether the inhibition of NF-κB activity mediated by cinacalcet occurred through the suppression of this signaling pathway. Results showed that cinacalcet could inhibit the phosphorylation of PKCδ, ERK, and P65 at 15, 60, and 30 min, respectively (Figures 6C–F). Collectively, cinacalcet suppressed the PKCδ/ERK/P65 signaling pathway.

As cinacalcet is a calcimimetic type Ⅱ compound and its typical target is CaSR, we used a selective antagonist of CaSR, NPS-2143, to test whether CaSR signaling played a role in the production of inflammatory cytokines. Inhibition of CaSR did not affect the mRNA expression and release of IL-6, whereas the mRNA expression and release of IL-1β were partially decreased, and cinacalcet could further reduce the production of IL-1β (Figures 6G–J). We further used an NF-κB inhibitor, JSH-23, to test the effect of NF-κB inhibition on the production of inflammatory cytokines. Results showed that the mRNA expression and release of IL-1β and IL-6 were significantly reduced by JSH-23, and cinacalcet could further reduce the production of IL-1β but not IL-6 (Figures 6G–J). Conclusively, the inhibitory effect of cinacalcet on the production of IL-6 is dependent on PKCδ/ERK/P65 signaling, whereas the inhibitory effect of cinacalcet on the production of IL-1β is partially dependent on the PKCδ/ERK/P65 signaling pathway. The possible mechanism explaining the anti-TNF activity of cinacalcet, by directly targeting NK1R, is summarized in a proposed model (Figure 6K).

## Discussion

Cinacalcet is traditionally used to treat primary and secondary hyperparathyroidism. It has also been investigated for the treatment of hyperparathyroidism-associated diseases, including familial hypophosphemic rickets, recurrent prostate cancer, osteoporosis, and renal osteodystrophy. Our study reported that cinacalcet inhibited production of the inflammatory cytokines TNFα, IL-1β, and IL-6, via suppression of the PKCδ/ERK/P65 signaling pathway, by targeting NK1R, indicating that cinacalcet might be repurposed for use in treating IBD.

In the inflammatory environment of IBD, TNFα can be produced by several cell types, including macrophages, dendritic cells, T cells, adipocytes, and fibroblasts, and TNFα plays a critical role in the pathogenesis of IBD (Neurath, 2014; Wallace et al., 2014). After TNFα binding to TNFα receptors Ⅰ and Ⅱ, the NF-κB signaling pathway is activated, leading to translocation of the transcription factor NF-κB P65 from the cytoplasm to nucleus, ultimately causing various pro-inflammatory effects. These effects include the induction of angiogenesis, causing the death of Paneth cells via necroptosis, and promoting the production of matrix metalloproteinases by myofibroblasts that originated from stromal cells; TNFα can also activate macrophages and effector T cells and directly damage intestinal epithelial cells (Meijer et al., 2007; Günther et al., 2011; Neurath, 2014). Furthermore, activation of the NF-κB signaling pathway by TNFα results in positive feedback to induce production of the inflammatory cytokines TNFα, IL-1β, IL-6, and IL-23 by macrophages. These cytokines can induce expression of the transfection factor T-bet and RORγt in T cells, thus promoting differentiation from naïve T cells to Th1 and Th17 cells (Wallace et al., 2014). Therefore, inhibiting the TNFα-stimulated NF-κB signaling pathway exerts anti-inflammatory effects.

As macrophages play an important role in the pathogenesis of IBD, they have been considered treatment targets (Bain and Mowat, 2014; Na et al., 2019). In the pathogenesis of IBD, macrophages, which are monocyte-like cells, promote inflammation by aggregating in the inflamed colon and secreting large amounts of IL-1β, IL-6, TNFα, and IL-23 (Kamada et al., 2008). Differentiation from monocytes to macrophages is altered in IBD, resulting in an abnormal macrophage morphology with CD14^hi^ expression. This weakens the ability to clear bacteria, prolonging bacterial survival (Smith et al., 2009). IL-1β and IL-6 also play crucial roles in the pathogenesis of IBD (Coccia et al., 2012; Yao et al., 2014) by promoting the production of inflammatory cytokines via activation of the NF-κB pathway. Additionally, IL-1β can act in concert with IL-6 to induce the differentiation of naïve T cells to Th17 T cells. Thus, biologic agents targeting IL-1β and IL-6 might also be effective for treating IBD (Coccia et al., 2012; Yao et al., 2014; Mao et al., 2018). Considering the critical roles of macrophages and cytokines in IBD pathogenesis, we used macrophages to assess the anti-inflammatory effects of cinacalcet and found that it could inhibit the production of pro-inflammatory cytokines.

The transcription factor NF-κB plays a crucial role in immune system regulation, and its aberrant activation is correlated with various inflammatory diseases including IBD. Therefore, targeting NF-κB activation is effective for treating autoimmune diseases (O'Sullivan et al., 2007; Li and Verma, 2002). The translocation of NF-κB P65 from the cytoplasm to the nucleus indicates its activation. We found that cinacalcet inhibited the translocation of NF-κB P65. Thus, cinacalcet was effective for treating DSS-induced colitis by inhibiting the activation of NF-κB induced by TNFα.

Previously published studies reported that the pathological role of TNFα in inflammation, via NK1R, includes two aspects. On one hand, TNFα could induce the expression of NK1R (McKinnon et al., 2013). On the other hand, TNFα could promote macrophages and intestinal neurons to synthesize p substance, an endogenous ligand of NK1R. Thus, after increased p substance binding to NK1R, phospholipase C will be activated, and this protein degrades phosphatidylinositol 4,5-bisphosphate to form inositol 1, 4, 5-triphosphate and diacylglycerol, which are the second messengers that stimulate calcium mobilization and protein kinase C activation, leading to the phosphorylation of ERK and NF-κB activation. After the translocation of NF-κB P65 to nuclei, inflammatory cytokines, such as TNFα, IL-1β and IL-6, will be produced (Spitsin et al., 2018; Koon and Pothoulakis, 2006; O’Connor et al., 2004). Correspondingly, cinacalcet can prevent PKCδ/ERK/P65 signaling by targeting NK1R.

Although cinacalcet is an agonist of CaSR, blocking CaSR with NPS-23 did not affect production of the inflammatory cytokine IL-6, which suggested that cinacalcet targeted a new molecule. By target prediction, we found the new target of cinacalcet, NK1R, and its downstream signaling pathway involved in inflammation. Nevertheless, production of the inflammatory cytokine IL-1β was partially affected by blocking CaSR, which could be explained by the fact that CaSR could active NLRP3 (Lee et al., 2012; Ren et al., 2020), a key regulator of IL-1β secretion (Grebe et al., 2018). However, the mechanism through which CaSR activates NLRP3 in colitis needs further study.

In summary, we find a new target of cinacalcet, NK1R, which is different from the already known target CaSR. We also show that cinacalcet exerts anti-inflammatory effects by inhibiting the PKCδ/ERK/P65 signaling pathway. This provides theoretical support for treating DSS-induced colitis and diseases in which NF-κB activation plays a critical pathogenic role.

## Data Availability

The original contributions presented in the study are included in the article, further inquiries can be directed to the corresponding authors.
